# Were Equatorial Regions Less Affected by the 2009 Influenza Pandemic? The Brazilian Experience

**DOI:** 10.1371/journal.pone.0041918

**Published:** 2012-08-01

**Authors:** Cynthia Schuck-Paim, Cécile Viboud, Lone Simonsen, Mark A. Miller, Fernanda E. A. Moura, Roberto M. Fernandes, Marcia L. Carvalho, Wladimir J. Alonso

**Affiliations:** 1 Origem Scientifica, São Paulo, São Paulo, Brazil; 2 Division of International Epidemiology and Population Studies, Fogarty International Center, National Institutes of Health, Bethesda, Maryland, United States of America; 3 School of Public Health and Health Services, George Washington University, Washington, District of Columbia, United States of America; 4 Virology Laboratory, Pathology and Medicine Department, Universidade Federal do Ceará, Fortaleza, Ceará, Brazil; 5 General Coordination of Epidemiological Information and Analyses, Brazilian Ministry of Health, Brasília, Distrito Federal, Brazil; 6 General Coordination of Infectious Diseases, Health Surveillance Secretariat, Brazilian Ministry of Health, Brasília, Distrito Federal, Brazil; University of Hong Kong, Hong Kong

## Abstract

Although it is in the Tropics where nearly half of the world population lives and infectious disease burden is highest, little is known about the impact of influenza pandemics in this area. We investigated the mortality impact of the 2009 influenza pandemic relative to mortality rates from various outcomes in pre-pandemic years throughout a wide range of latitudes encompassing the entire tropical, and part of the subtropical, zone of the Southern Hemisphere (+5^°^N to −35^°^S) by focusing on a country with relatively uniform health care, disease surveillance, immunization and mitigation policies: Brazil. To this end, we analyzed laboratory-confirmed deaths and vital statistics mortality beyond pre-pandemic levels for each Brazilian state. Pneumonia, influenza and respiratory mortality were significantly higher during the pandemic, affecting predominantly adults aged 25 to 65 years. Overall, there were 2,273 and 2,787 additional P&I- and respiratory deaths during the pandemic, corresponding to a 5.2% and 2.7% increase, respectively, over average pre-pandemic annual mortality. However, there was a marked spatial structure in mortality that was independent of socio-demographic indicators and inversely related with income: mortality was progressively lower towards equatorial regions, where low or no difference from pre-pandemic mortality levels was identified. Additionally, the onset of pandemic-associated mortality was progressively delayed in equatorial states. Unexpectedly, there was no additional mortality from circulatory causes. Comparing disease burden reliably across regions is critical in those areas marked by competing health priorities and limited resources. Our results suggest, however, that tropical regions of the Southern Hemisphere may have been disproportionally less affected by the pandemic, and that climate may have played a key role in this regard. These findings have a direct bearing on global estimates of pandemic burden and the assessment of the role of immunological, socioeconomic and environmental drivers of the transmissibility and severity of this pandemic.

## Introduction

Novel influenza virus with pandemic potential emerge every few decades, and the fear of rapid global transmission of a deadly pathogen, as experienced in the tragic influenza pandemic of 1918, has shaped research and public health policies in this area. In late April 2009, a novel swine-origin A/H1N1 influenza pandemic virus was identified in Mexico and the US [1] and disseminated globally within weeks [2]. By mid-June, there were over 35,000 cases reported in more than 70 countries and nearly two hundred laboratory-confirmed deaths [2], especially among younger adults, prompting the World Health Organization to declare the first influenza pandemic in over 40 years [3].

Despite successful efforts to characterize the epidemiology of this pandemic in temperate areas of the Southern Hemisphere [4], [5], [6], [7], [8], [9], little is known about spatial patterns of pandemic disease impact and dynamics in the tropical zone. The drivers of influenza seasonality in inter-pandemic periods have remained a long-standing enigma in the Tropics [10], and even less is known about pandemic patterns in this region. Yet it is in the tropics where nearly half of the world population lives, where infectious disease burden is highest, and where access to proper health care systems is most frequently lacking.

Brazil offers a unique opportunity to investigate spatio-temporal patterns in pandemic dynamics and impact in a large population dispersed across a wide range of tropical and subtropical latitudes, yet exposed to relatively uniform health care and surveillance systems, immunization policies, as well as to the same preparedness and mitigation efforts. In this study we characterize the mortality impact and dissemination patterns of the 2009 influenza pandemic across Brazil and investigate the role of geographic, demographic, and economic factors. We find evidence of a robust latitudinal gradient, spanning over 40° of latitude, both in the onset of pandemic-associated mortality and in the mortality impact of the pandemic relative to pre-pandemic years that was independent of socio-demographic factors and inversely related with income. Additionally, our findings suggest a key role of climate on the spatio-temporal dynamics of the pandemic in this region of the world, and indicate that the mortality burden of the 2009 pandemic in the Tropics may have been substantially lower than currently suggested.

## Materials and Methods

### Mortality and Demographic Data

We analyzed publicly available mortality datasets from two independent sources: vital statistics maintained by the Brazilian Ministry of Health (1996–2010) and laboratory-confirmed pandemic A/H1N1pdm deaths compiled by Brazil’s National Surveillance Information System of Notifiable Diseases (SINAN) as of March 2012.

#### Vital statistics

Monthly vital statistics from the Mortality Information System (SIM) of the Brazilian Ministry of Health [11] were aggregated by state (27 administrative units: 26 states and the Federal District), age (<5, 5–14, 15–24, 25–44, 45–64, and >64 years), and cause of death (as coded by ICD10; [12]). We included data going back to 1996 to allow for estimation of a pre-pandemic baseline against which to compare pandemic-related mortality patterns. Considering previous work showing that pneumonia and influenza (hereafter P&I) deaths are the most specific endpoint for studying influenza-related mortality [13], [14], we focused our analysis on P&I deaths (ICD10: J09–J18.9). We also analyzed deaths from sensitive outcomes, including all deaths from respiratory (ICD10: J-coded deaths) and circulatory (ICD10: I-coded deaths) causes, as these outcomes have been linked to influenza mortality [15]. Vital statistics are uniformly and systematically collected throughout the year and covered approximately 93% of the population in 2008 [11]. Regional differences in coverage ranged from 83.1% in the Northern Region to 96.7% in Southeastern Region (data available for 2008; [11]). The only state where coverage was 100% was the Federal District, in the Central Western region of Brazil. Although small, these differences were taken into account to adjust the estimates of pandemic-associated mortality (see ‘Data Analysis’ for details).

#### Laboratory-confirmed deaths

In Brazil, notification and testing of suspected pandemic A/H1N1pdm influenza cases and deaths was mandatory [16], [17]. Initially, case-definition was restricted to inpatients and outpatients presenting with fever higher than 38°C, cough, and close contact with an infected person or recent travel to countries with confirmed cases. However, as transmission became widespread (after epidemiological week 28 of 2009, July 16), mandatory notification and laboratory investigation were restricted to all patients presenting with severe acute respiratory infections (SARI), including fever, cough, and dyspnoea or death [17]. Nationwide notification of cases and deaths was made through a national web-based reporting system. Respiratory specimen collection and diagnosis was performed by the National Influenza Surveillance System, a network of 62 sentinel units established in 2000 to monitor virus circulation systematically in all Brazilian states. Specimen collection was standardized throughout the country and testing of respiratory specimens for A/H1N1pdm by real-time RT-PCR was centralized at three reference laboratories (Instituto Adolfo Lutz; Instituto Evandro Chagas; Fundação Oswaldo Cruz) [17].

#### Population Estimates

Population estimates for each state and age group, demographic density and the proportion of the population in urban areas were obtained from the Brazilian Institute of Geography Statistics (IBGE; censuses 1993, 2001 and 2010). Annual population data were calculated by spline interpolation of census data.

#### Data analysis

Our analysis focused on quantifying pandemic mortality burden relative to pre-pandemic mortality in the first year of A/H1N1pdm circulation in each Brazilian state and identifying state-specific timing of the onset of pandemic-associated mortality. Pandemic-related mortality was estimated for the period from June 1, 2009 to May 30, 2010, covering a full year of pandemic virus circulation. However, the mortality datasets were also inspected until the end of 2010 to enable the detection of subsequent pandemic waves.

Vital statistics series were used to estimate the anomalous mortality burden caused by the pandemic, which we define as the mortality levels observed during the pandemic year above those expected over the entire course (including all seasons) of an average non-pandemic year. Laboratory-confirmed data provided a measure of the mortality burden specific to the A/H1N1 pandemic strain, with the caveat that such data is likely to underestimate mortality, as it may be prone to detection biases and does not capture deaths caused by secondary complications, when viral detection is no longer possible. Laboratory-confirmed pandemic deaths were also used to define the onset of A/H1N1pdm mortality in each Brazilian state.

Expected mortality in the absence of a pandemic was determined for each state based on monthly mortality data from pre-pandemic years (1996–2008) by using a Serfling-type (periodic) regression model [18] that did not involve the a priori definition and subsequent exclusion of epidemic seasons. The baseline model was then extrapolated to the pandemic period (2009–2010) and compared with observed mortality in the pandemic months. This modeling process involved three steps. First, we calculated a secular trend in mortality by fitting a linear or quadratic polynomial (the latter when model fit increased by at least 3%) that maximized the fit between observations and model. We then estimated pre-pandemic (1996–2008) seasonal variation by decomposing the de-trended time series into its constituent Fourier series of harmonics. The model can be described formally as:


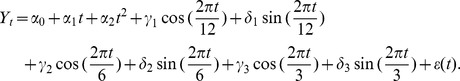


where Y_t_ is mortality at month *t*, α_1_ *t* and α_2_ *t^2^* the linear and quadratic secular trends, *γ* and *δ* the regression coefficients of the periodic (annual, semi-annual and quarterly) components and ε(t) a normally-distributed error term. By summing up the periodic components and the secular trend, we obtained a seasonal-trend model (baseline) for the original series. Finally, to distinguish between epidemiologically significant departures from expected (pre-pandemic) mortality levels and year-to-year random variations [18] the model incorporated a 95% confidence interval, so only values lying outside this interval in the pandemic period were considered to determine the additional pandemic mortality. Model residuals were visually inspected to ensure that seasonality and trends had been appropriately removed.

It is important to note that the methodology employed here to calculate baseline mortality does not discard those deaths from pre-pandemic periods of seasonal influenza activity, and quantifies pandemic mortality burden influenza as that above and beyond the burden of seasonal epidemics in each state. Excess pandemic mortality thus represents the ‘anomalous’ mortality during the pandemic (after taking into account trend, seasonality and usual variability from previous years), and not necessarily the total mortality burden of the A/H1N1 pandemic strain. Additionally, because mortality in the pandemic period is compared to that in pre-pandemic years within a same state, potential differences in reporting levels among states are not relevant for our analyses because there is no reason to expect that vital statistics collection would be geographically biased only in the pandemic year. In those cases where these differences are relevant (for absolute estimates of additional pandemic deaths), we adjusted our estimates in each state by using official underreporting estimates for each state [11]. In addition, we also calculated the monthly z-scores of the additional pandemic mortality (representing the number of standard deviations above baseline observed during the pandemic period in each state) as a further standardized measured of pandemic mortality to be compared among states.

### Spatiotemporal Analysis

The onset of laboratory-confirmed pandemic deaths and the estimates of pandemic-associated mortality were analyzed in relation to the latitude of the state capital. Because the relationship between latitude and mortality can be confounded by social and demographic factors that also vary with latitude in Brazil (Table S1), we compiled data on population size, density, the ratio of young adults to seniors (15–55 years of age vs. > = 65 years), proportion of the population living in rural areas, and distance from the state where the first laboratory-confirmed death was reported. The latter factors were analyzed in general linear models adjusting for latitude. Analyses were conducted using Epipoi (http://www.epipoi.info, a software for the analysis of epidemiological times series written with Matlab R2007 scripts by WJA), and IBM SPSS Statistics v.17. *P*-values are two-tailed.

## Results

### National Pandemic Mortality Patterns

**Figure 1:**
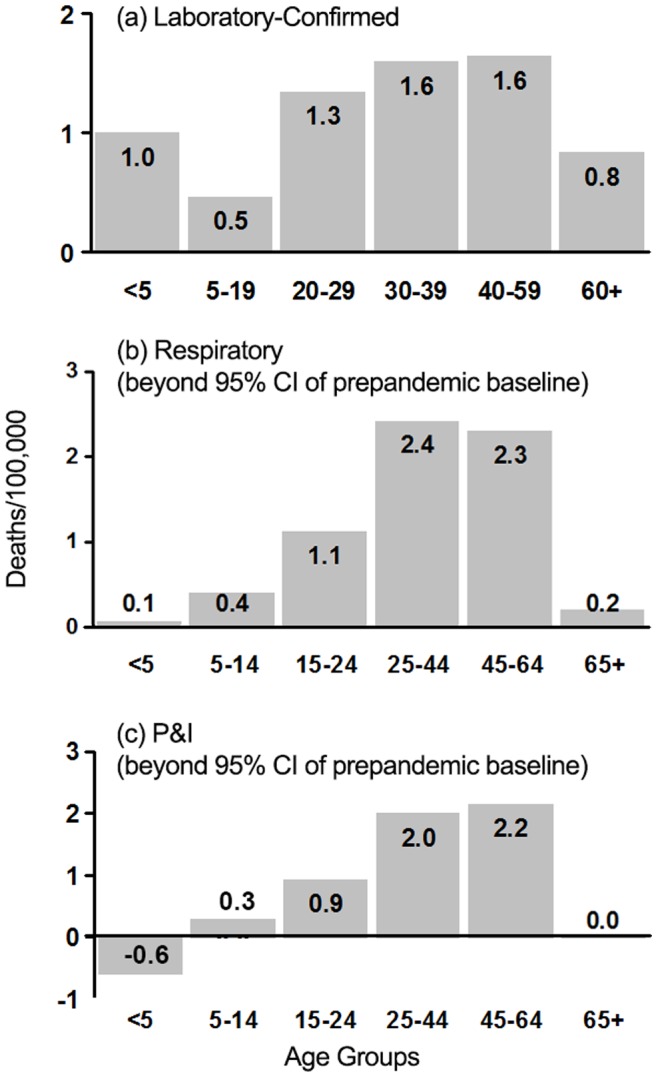
Age-specific mortality rates in Brazil (May 2009– Apr 2010) based on laboratory-confirmed pandemic deaths and mortality from vital statistics. Note that respiratory and P&I mortality rates (representing the sum of residuals outside the 95% C.I. defining baseline mortality in pre-pandemic years) capture the impact of the pandemic beyond that of annual epidemics, and do not represent total mortality of the 2009 A/H1N1 pandemic strain. There were no excess deaths from circulatory causes.

The pandemic surveillance system captured 2,179 laboratory-confirmed deaths from late May 2009 (week 21) to May 2010 (week 21), corresponding to a laboratory-confirmed mortality rate of 1.15 deaths per 100,000 inhabitants. The highest death rates were among adults aged 25–64 years, as measured by laboratory-confirmed data, as well as by the respiratory and P&I deaths above baseline levels in non-pandemic years (Fig.1). All measures consistently indicated that seniors over 65 years were largely spared (Fig.1). The rate of laboratory-confirmed mortality in young children, particularly newborns (<1 year), was high, although this was not seen in excess P&I and respiratory deaths.

**Figure 2:**
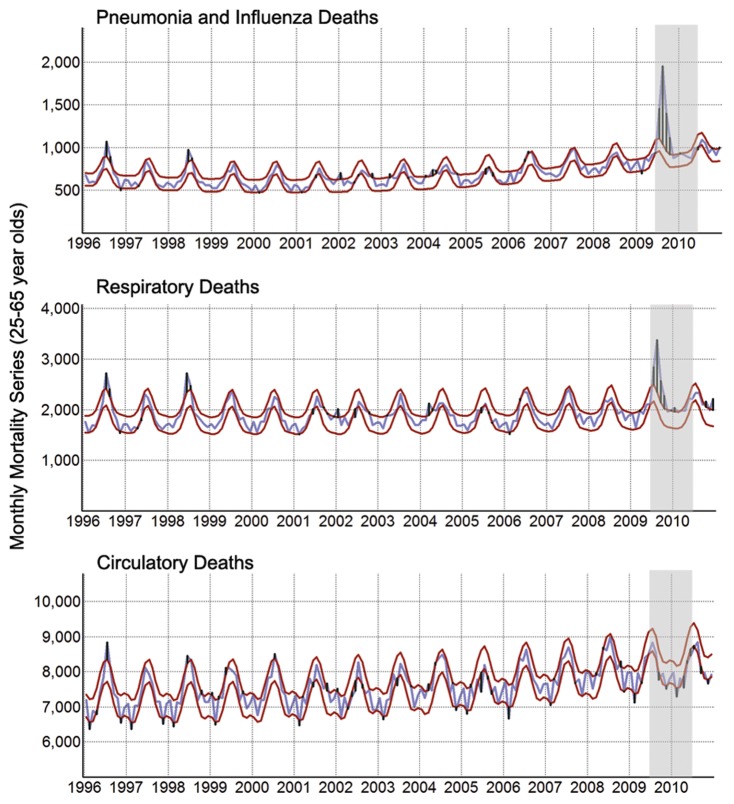
Monthly mortality series, Brazil, 1996–2010. The blue line represents observed mortality among adults aged 25–55 years and the red lines represent the 95% CI of the seasonal-trend mortality model calculated from 1996 to 2008, and extrapolated up to 2010. Positive and negative residuals (black bars) represent positive and negative differences between the data and the 95% CI. Excess mortality was calculated as the sum of residuals within the pandemic period (shaded area).

There were 2,273 and 2,787 P&I- and respiratory-associated deaths in Brazil during the pandemic in addition to those expected over the entire course of an average year (∼1.2 and 1.5 additional deaths/100,000, respectively), corresponding respectively to a 5.2% and 2.7% annual increase over average pre-pandemic mortality levels (average of approximately 43,500 and 103,000 annual P&I and respiratory deaths, respectively, from 2006 to 2008). Figure 2 shows the mortality series for the most affected age groups. The spike in mortality coincided with the pandemic period (shaded). There was no additional mortality in 2009 or 2010 from circulatory (Fig.2), cerebrovascular, and ischemic heart diseases (data not shown).

### Progressively Milder Mortality Impact in Lower Latitudes

**Figure 3:**
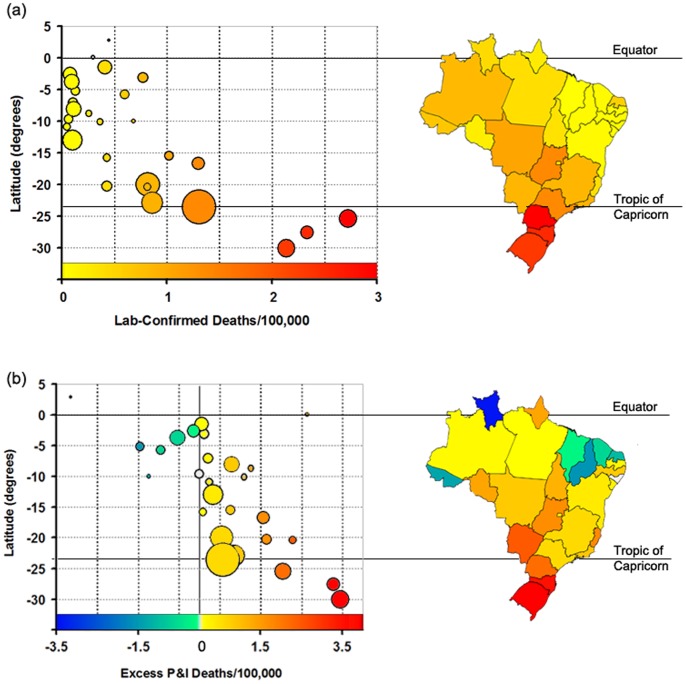
Latitudinal gradient in pandemic-associated mortality in Brazil. Laboratory-confirmed pandemic death rates (a) and P&I mortality rates in excess of pre-pandemic baseline mortality (including seasonal epidemics) (b) from June 01, 2009 to May 30, 2010 in each state (all age groups). The size of the data points is proportional to the population of each state. The same colors are used to show each state on the graph and map.

There was a consistent latitudinal gradient in laboratory-confirmed mortality rates and the anomalous levels of P&I mortality during the pandemic year (Fig.3), with pandemic impact gradually decreasing from the South to the North Brazil (regression of 2009–2010 death rate against latitude, *p* = 0.001, *r* ^2^_adj_ = 0.38 for laboratory confirmed deaths and *p*  = 0.001, *r* ^2^_adj_ = 0.42 for additional P&I deaths). The analysis was also significant when restricted to 2009 only (Box S1). Additionally, a significant association between the mean z-scores of pandemic mortality in each state during 2009–2010 and latitude was observed (*F_1,25_* = 7.8, *p*  = 0.01, *r* ^2^_adj_  = 0.21), confirming the observation that lower latitude states (closer to the equatorial region) experienced lower deviation from baseline mortality during the pandemic period. Visual inspection of Fig.3 suggested that the relationship was stronger in those states below latitude 15°S. Indeed, there was a strong latitudinal gradient in pandemic-associated death rates below 15°S (*r* ^2^_adj_ = 0.43, *p* = 0.017 for laboratory-confirmed deaths and *r* ^2^_adj_ = 0.49, *p* = 0.010, for P&I mortality), but not for the states above it (*p*  = 0.18 and *p* = 0.32, respectively). The analysis of all respiratory outcomes (all ICD10 J-coded deaths) also confirmed the presence of a latitudinal gradient in pandemic-related respiratory mortality during the pandemic year (*F_1,26_* = 4.96, *p*  = 0.03, *r* ^2^_adj_ = 0.13).

Next, we assessed whether the observed gradient in pandemic mortality may be confounded by social and demographic factors. We modeled mortality rates as a function of latitude, after controlling for the effect of population size, demographic density, proportion of the population living in urban areas, distance from the presumed start of the pandemic and age structure of the population (Box S2). The effect of latitude on mortality remained significant in all models and explained the largest proportion of variance in state-specific laboratory-confirmed influenza mortality rates and pandemic-associated P&I mortality rates (Tables S2 and S3). We also investigated the possibility that the A/H1N1pdm virus became less virulent over time, so that those states hit earliest experienced highest mortality. However, the association between latitude and pandemic mortality remained significant after adjusting for differences in the timing of pandemic onset, thus weakening this hypothesis (*r* ^2^_adj_ = 0.35, *p* = 0.004 for laboratory-confirmed deaths and *r* ^2^_adj_ = 0.44, *p*<0.001, for pandemic-associated P&I deaths).

### Progressively Later Onset of Pandemic-associated Mortality in Lower Latitudes

**Figure 4:**
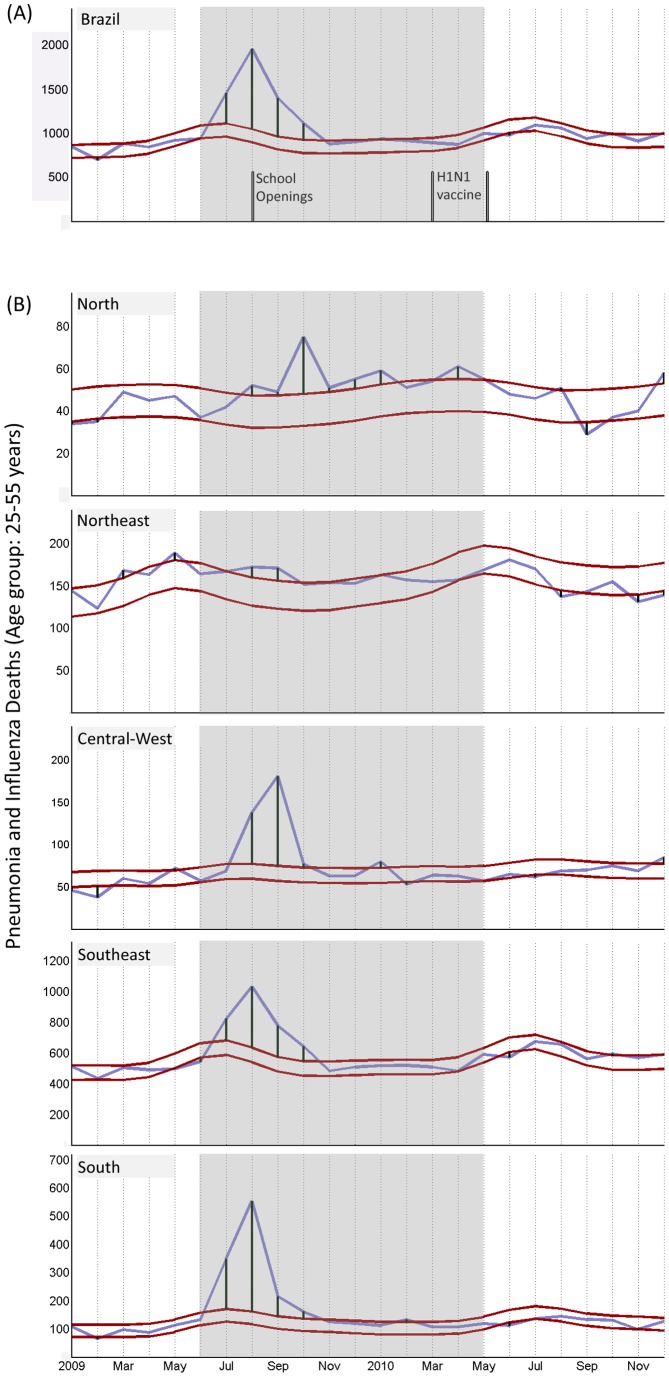
Pneumonia and Influenza mortality. Pneumonia and influenza deaths in adults aged 25–55 years in Brazil (A) and each Brazilian region (B). Schools opened on August 3 (2009) in most states. Red and blue lines represent, respectively, the 95% C.I. of the baseline mortality (including seasonal epidemics) of pre-pandemic years and observed mortality. The shaded area represents the 12 first months of pandemic virus circulation. A/H1N1pdm vaccines were distributed in 5 phases: Mar 8 (health workers and indigenous populations), Mar 22 (pregnant women, children <6 months and those with chronic conditions), Apr 5 (age group: 20–29 years), Apr 24 (≥60 years); May 10 (age group: 30–39 years).

**Figure 5:**
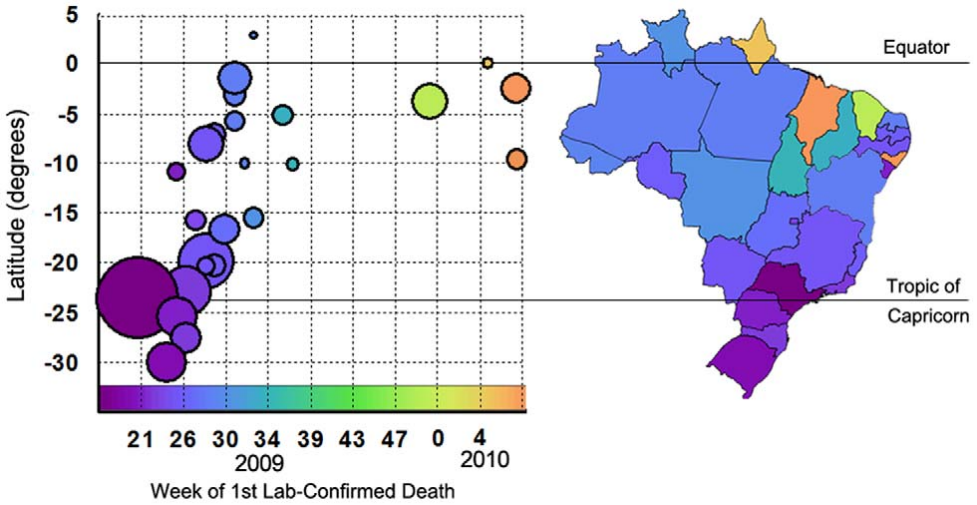
Geographical patterns in timing of pandemic mortality in Brazil: week of the first lab-confirmed death by state. The size of the data points is proportional to the population of each state. The same colors are used to show each state on the graph and map.

**Figure 6:**
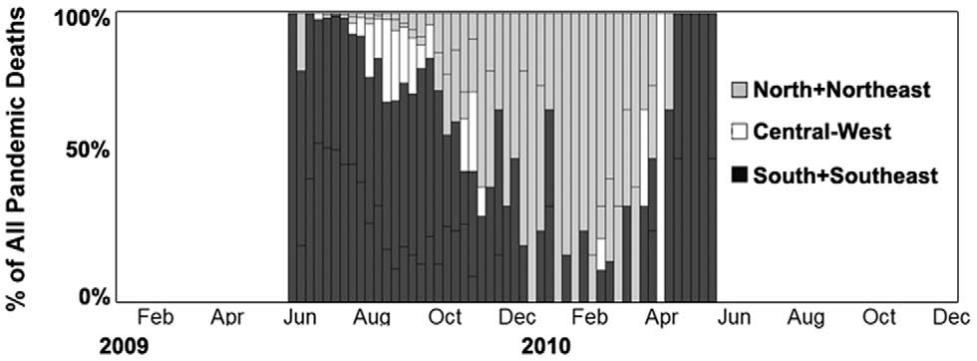
Weekly percentage of laboratory-confirmed A/H1N1pdm deaths by Brazilian region.

The analysis of national pandemic-associated P&I mortality data including all age groups, as well as the most affected age groups (25–55 years), demonstrated that pandemic mortality peaked approximately in August in the Southern Hemisphere winter season (Fig.4A). Mortality aggregation at the country level hides, however, important geographical differences in pandemic dynamics. Accordingly, the analysis of pandemic-associated mortality onset in each state, as measured by laboratory-based surveillance data, revealed a robust latitudinal gradient across Brazil, with a Northward progression in pandemic timing in 2009 (*F* = 15.1, *p = *0.001, *r* ^2^ = 0.40) (Fig.5). Also, some states of the coastal northern and northeastern states in low latitude regions did not report laboratory-confirmed deaths until 2010 (yellow and orange circles in Fig.5, see also map). A Northward progression of pandemic mortality was also evident if these states were included in an analysis of pandemic onset during 2009–2010 (*r* ^2^ = 0.33, *p* = 0.001). Similarly, the latitudinal gradient in pandemic onset was maintained when the analysis was limited to the period after local transmission became widespread (change in case-definition from week 28 onwards, *F* = 8.7, *p* = 0.007, *r* ^2^ = 0.26). These patterns were additionally confirmed by the analysis of P&I mortality from vital statistics, which showed that peak times in pandemic-associated mortality were earliest in the South of Brazil (Fig.4B). Further, the analysis of all laboratory-confirmed deaths showed that most deaths in 2009 occurred in the Southern and Southeastern states, coinciding with the winter in those regions (Fig.6). In contrast, deaths identified later in 2009 and 2010 occurred predominantly in the Northern and Northeastern states, coinciding with the rainy season in these regions (Fig.6).

## Discussion

Our investigation of the spatio-temporal patterns in pandemic dynamics in a large population exposed to uniform immunization and mitigation policies and dispersed across a broad region encompassing the entire tropical, and part of the subtropical, zone of the Southern Hemisphere revealed a robust spatial pattern in mortality, with progressively lower and later pandemic-related mortality impact near equatorial areas. The patterns observed were consistent for two independent state-specific datasets, including laboratory-confirmed influenza deaths and pandemic-related mortality derived from a time series model of both specific (P&I) and sensitive (respiratory) outcomes, lending support to our conclusions. These findings are of relevance for understanding the impact of the 2009 influenza pandemic in Brazil and, if confirmed in other equatorial zones, can also affect estimates of pandemic impact globally (which so far assumed similar or even higher burden in tropical and low income regions; [19]), as well as the assessment of the relative role of immunological and environmental factors on the transmissibility and severity of this pandemic.

To our knowledge this is the first study reporting both a robust latitudinal gradient in pandemic mortality and timing and consistently milder pandemic mortality impact in equatorial regions, where little or no difference from pre-pandemic mortality levels was observed. Several factors make it unlikely that such gradients stemmed from differences in reporting and collection efforts among states or from methodological biases. First, the geographic patterns were independent of data sources, study period and type of analysis employed. Particularly, the association between the mortality impact of the pandemic and latitude was strongest in those regions for which the national vital statistics system had best coverage (between latitudes 15–30°S, where coverage was virtually 100%; [11]). The results were also consistent when the analysis was restricted to the period when laboratory investigation was restricted to cases of severe acute respiratory infection, therefore being independent changes in case definition over the course of the period studied. The validation of the results through the analysis of all respiratory deaths, in addition to P&I, additionally speaks against the possibility that the higher pandemic-associated mortality in high latitude areas derived from diagnostic shifts within the respiratory group. The independent validation of the results by laboratory surveillance data also makes it unlikely that they stemmed from putative coding changes. Importantly, coding deficiencies would have to be geographically structured to produce the spatiotemporal patterns observed, a possibility we find unlikely.

Unlike previous efforts, the modeling approach employed here measured pandemic burden relative to typical annual mortality levels within each location (as opposed to absolute pandemic mortality rates of the A/H1N1 pandemic strain). Accordingly, the calculation of baseline mortality included all P&I and respiratory deaths from pre-pandemic years, and did not require the discrimination between those potentially attributable to influenza and to other causes. Incorporating the usual inter-annual variability in the complete mortality series in each location in the baseline model (including that of seasonal epidemics) also made the model suitable for the comparison of mortality across regions with differing seasonality (including tropical areas and regions lacking clearly defined season), as it did not require the a priori definition of epidemic and non-epidemic seasons. Additionally, the analysis was not subject to issues inherent to differentiating influenza-related mortality from deaths caused by other illnesses or pathogens in pre-pandemic years, or with establishing pre-pandemic differences in seasonal influenza burden or the extent of pandemic strain replacement across regions. Here, baseline mortality and its corresponding variability in each state acted as its own control for interstate biases deriving from epidemiological, seasonal or socio-economic confounders.

In this study, the latitudinal gradient in pandemic-associated mortality reported was consistent even after controlling for factors previously associated with the transmissibility of the pandemic [4], [20], including population size, density, age structure, level of urbanization and distance from the presumed start of the pandemic. Interstate differences in mortality could have alternatively derived from economic differences, as better nutrition, health and access to nursing care could translate into lower mortality. However, the lowest burden estimates were in the low income states in the Northeast and North regions, whereas much higher mortality levels were observed in the rich states in the Southeast and South regions. The inverse association between latitude and income in Brazil [21] in this sense speaks against the hypothesis of a prevailing role of low income towards a higher risk of pandemic-associated deaths throughout Brazil. In addition, differences in vaccination and antiviral administration cannot explain geographical differences in pandemic-associated mortality, as in Brazil mitigation efforts were coordinated at a national level and the A/H1N1pdm vaccine was distributed at the end of the second pandemic wave in 2010 [22] (Fig.4A). Overall, our results thus suggest that the observation of a hardly burdened south and milder mortality burden nearer the equatorial region may be associated with regional differences in environmental factors that modify the transmissibility or severity of pandemic-related infections [23], as similarly suggested for seasonal influenza in Brazil [24]. The notion that environmental factors, chiefly climate, may underlie the reported differences in burden is also indicated by the observation of differences in pandemic-associated mortality within the equatorial region itself, as northeastern states were in general characterized by a lower burden than the northern states (Fig.3 and 4B). Considering that influenza epidemics tend to be associated with the rainy season in the low latitude areas of Brazil [25], this pattern might be related to the fact that rainy seasons in the Northern states are longer and more intense than in the Northeastern region of Brazil, where the climate is drier.

Pandemic-related mortality during the first year of circulation of the A/H1N1pdm peaked approximately in August 2009, coinciding with the Southern Hemisphere winter. Although school closures have been associated with reduced influenza transmission [4], [20], [26], school holidays were relatively uniform throughout Brazil, starting in most states in the first two weeks of July 2009 and ending in the beginning of August 2009. Therefore school patterns are an unlikely driver of these spatio-temporal mortality trends. Conversely, the observation that the peak of pandemic mortality occurred in August 2009 in the Southern states, and progressively later with milder impact towards the North of Brazil echoes the usual timing of seasonal influenza mortality across Brazil [24], whereby Southern Brazil experiences influenza activity during the temperate Southern Hemisphere winter (June–September) and tropical Northern States experience seasonal influenza mortality in the rainier months (early in the year). Given the very long time lag between the onset of fatal pandemic cases in Southern and Northern Brazil (34-week between the earliest and latest states to report A/H1N1pdm fatalities), it is unlikely that these patterns can be fully explained by differences in population movement and connectivity between Brazilian states, or from geographical differences in transmissibility [27]. This reinforces the suggestion that climate was a major driver of the spatiotemporal patterns of pandemic mortality in Brazil, in line with evidence pointing to a link between temperature, humidity and the transmissibility of the 2009 pandemic strain [28].

Although our results did not support the possibility that the A/H1N1pdm virus became less virulent over time, it would be possible that mortality in the first year of A/H1N1 circulation was low, and that a second wave of mortality occurred later in the equatorial regions of Brazil given the later onset in pandemic-associated mortality. The inspection of both P&I and respiratory mortality until the end of 2010 showed, however, that this was not the case (Fig.4): mortality levels were also low throughout 2010, further confirming the notion that equatorial regions of Brazil were less affected by the 2009 pandemic. Further research is, however, needed to determine if the finding of lower pandemic burden in tropical climates can be generalized elsewhere. In a study which found evidence of a possible lower pandemic burden in tropical areas of Africa [29], the authors interpret this finding as being likely due to underreporting [29]. In fact, a recent study estimated that most global pandemic deaths may have occurred in Africa and southeast Asia, yet no direct data on respiratory mortality was available for these areas [19]. Although there is no question about the importance of respiratory infections in developing and tropical areas, the present study raises instead the possibility that pandemic mortality in tropical climates may have been substantially lower than currently suggested.

In contrast to mortality patterns observed in low latitude areas, subtropical and richer states of Southern Brazil were significantly and heavily burdened by the pandemic compared to previous years (in some cases, as in the state of Rio Grande do Sul, with over 3.6 P&I deaths/100,000 inhabitants above the baseline; rightmost circle on Fig.3b), particularly those individuals aged 25 to 65 years (Fig.4). This relatively young age distribution of pandemic-related deaths is consistent with that described in other settings, including Mexico and the US [30], [31], [32]. It is important to note that, because the objective of our time series modeling was to estimate pandemic impact above the seasonal mortality norm, pandemic-associated mortality rates derived from vital statistics reported here do not represent estimates of the total mortality burden of this H1N1pdm strain. There is compelling evidence that the pandemic virus rapidly replaced seasonal influenza viruses in the Southern Hemisphere [33], so strain replacement likely led to lower death rates from seasonal influenza during the pandemic period. This means that a fraction of deaths included in our baseline mortality model included H1N1pdm-associated deaths. Therefore, the absolute excess mortality rates reported likely underestimate the burden of the A/H1N1pdm virus in Brazil, and are not directly comparable with laboratory-confirmed death rates. Still, we believe that the present modeling approach is more telling of the atypical character of an influenza pandemic than the comparison of H1N1pdm mortality estimates including those deaths otherwise potentially attributable to seasonal influenza. Strain replacement is an usual phenomena in seasonal influenza dynamics, whereas the potentially higher impact of a pandemic in terms of increased morbidity, mortality and general disruption is what sets it apart from seasonal epidemics for planning and preparedness purposes, enabling the assessment of above average requirements of healthcare services and supplies and the responses needed to cope with abnormal surges of need for medical care.

Comparing disease burden as reliably as possible is critical in areas marked by competing health priorities and limited resources, as is the case of many countries in the equatorial region. In Brazil, pandemic impact rose with distance from the equator independently of social and demographic drivers, suggesting that environmental factors played a key role in driving pandemic impact and onset. We speculate that other regions of the Southern Hemisphere with tropical climates may have been also disproportionally less affected by the pandemic. However, the proposed linkage between pandemic impact, onset, latitude and climate was not obvious until now, so further studies are necessary to confirm our findings. We suggest that such spatial heterogeneities, which go against economic gradients in a broad region of the tropical and subtropical Southern Hemisphere, are considered in global models of pandemic burden.

## Acknowledgments

This research was conducted in the context of the MISMS Study, an ongoing international collaborative effort to understand influenza epidemiological and evolutionary patterns, led by the Fogarty International Center, National Institutes of Health. The authors are grateful to the Department of Vital Statistics from the Brazilian Ministry of Health for providing the mortality data.

## Supporting Information

Box S1 **Analysis of latitudinal gradient in pandemic-associated mortality in Brazil in 2009.**
(DOC)

Box S2 **Analysis of the effect socio-demographic indicators on pandemic-associated mortality in Brazil.**
(DOC)

Table S1 **Bivariate correlation matrix showing the direction and magnitude of the association between socioeconomic and demographic indicators and latitude in Brazil.** To standardize the distribution of residuals population size, demographic density and age structure were log-transformed. The proportion of the population living in urban areas and the proportion of children in the population were square-root arcsine transformed.
(DOC)

Table S2 **General linear models testing the effect of socio-demographic factors on pandemic-associated mortality in Brazil.** The sequential sum of squares is used in all models. In the first column (p-value^1^) the demographic indicator is the first term in the model, latitude is the second, the binary location factor is the third and the interaction between latitude and the binary indicator is the fourth term. In the second column (p-value^2^) the order of the first and second term is inverted. Significant p-values^1^ and p-values^2^ indicate, respectively, a significant effect of the factor on mortality before and adjusting for latitude.
(DOC)

Table S3 **Multivariate model testing the effect of latitude, age structure and demographic density on pandemic death rates that were laboratory-confirmed (** ***R*** **^2^_adj_ = 0.73) in Brazil.**
(DOC)

## References

[1] Center for Disease Control (2009) Swine Influenza A (H1N1) Infection in Two Children – Southern California, March–April 2009. MMWR 58: 400–402. 19390508

[2] World Health Organization (2009) Situation updates - Pandemic (H1N1) 2009. http://www.who.int/csr/disease/swineflu/updates/en/index.html. Accessed 2011 September 2..

[3] World Health Organization (2009) World now at the start of 2009 influenza pandemic. http://who.int/mediacentre/news/statements/2009/h1n1_pandemic_phase6_20090611/en/index.html. Accessed 2011 September 2..

[4] Opatowski L, Fraser C, Griffin J, de Silva E, Van Kerkhove M, et al (2011) Transmission characteristics of the 2009 H1N1 influenza pandemic: comparison of 8 Southern Hemisphere countries. PLoS Pathog 7. 10.1371/journal.ppat.1002225 PMC3164643 21909272

[5] Baker MG, Kelly H, Wilson N (2009) Pandemic H1N1Influenza: Lessons from the Southern Hemisphere. Eurosurveillance 14. 10.2807/ese.14.42.19370-en 19883551

[6] van Kerkhove M, Mounts A (2011 ) 2009 versus 2010 comparison of influenza activity in southern hemisphere temperate countries. Influenza Other Respi Viruses 5. 10.1111/j.1750-2659.2011.00241.x PMC5780652 21668684

[7] Van Kerkhove M, Mounts A, Mall S, Vandemaele K, Chamberland M, et al. (2011 ) Epidemiologic and virologic assessment of the 2009 influenza A (H1N1) pandemic on selected temperate countries in the Southern Hemisphere: Argentina, Australia, Chile, New Zealand and South Africa. Influenza Other Respi Viruses 20. 10.1111/j.1750-2659.2011.00249.x PMC5780666 21668677

[8] Bishop J, Murnane M, Owen R (2009) Australia's winter with the 2009 pandemic influenza A (H1N1) virus. N Engl J Med 361: 2591–2594. 19940287 10.1056/NEJMp0910445

[9] Libster R, Bugna J, Coviello S, Hijano DR, Dunaiewsky M, et al (2009) Pediatric hospitalizations associated with 2009 pandemic influenza A (H1N1) in Argentina. N Engl J Med. NEJMoa0907673. 10.1056/NEJMoa0907673 20032320

[10] Tamerius J, Nelson M, Zhou S, Viboud C, Miller M, et al (2010) Global influenza seasonality: reconciling patterns across temperate and tropical regions. Environ Health Perspect 119. doi:10.1289/ehp.1002383. 10.1289/ehp.1002383 PMC3080923 21097384

[11] DATASUS (2011) Vital Statistics Brazil. Brazilian Ministry of Health (http://tabnet.datasus.gov.br/cgi/idb2010/a1801b.htm). Accessed 2011 August.

[12] WHO (2007) ICD-10; International Statistical Classification of Diseases and Related Health Problems, 10th Revision, Version for 2007. (http://appswhoint/classifications/apps/icd/icd10online, Accessed 2009 March.

[13] Reichert TA, Simonsen L, Sharma A, Pardo SA, Fedson DS, et al (2004) Influenza and the winter increase in mortality in the United States, 1959–1999. Am J Epidemiol 160: 492–502. 15321847 10.1093/aje/kwh227

[14] Viboud C, Bjornstad ON, Smith DL, Simonsen L, Miller MA, et al (2006) Synchrony, waves and spatial hierarchies in the spread of influenza. Science 312: 447–51. 16574822 10.1126/science.1125237

[15] Thompson WW, Shay DK, Weintraub E, Brammer L, Cox N, et al (2003) Mortality associated with influenza and respiratory syncytial virus in the United States. JAMA 289: 179–186. 12517228 10.1001/jama.289.2.179

[16] Brazilian Ministry Health (2006) Mandatory Diseases Notification Act of 2006 Official Journal (portuguese). Available: http://portalsaudegovbr/portal/arquivos/pdf/portaria_5_2006.pdf.

[17] Oliveira W, Carmo E, Penna G, Kuchenbecker R, Santos H, et al (2009) Pandemic H1N1 influenza in Brazil: analysis of the first 34,506 notified cases of influenza-like illness with severe acute respiratory infection (SARI). Euro Surveill 14: 19362. 19883548 10.2807/ese.14.42.19362-en

[18] Serfling R (1963) Methods for current statistical analysis of excess pneumonia-influenza deaths. Public Health Rep 78: 494–506. 19316455 PMC1915276

[19] Dawood F, Iuliano A, Reed C, Meltzer M, Shay D, et al. (2012 ) Estimated global mortality associated with the first 12 months of 2009 pandemic influenza A H1N1 virus circulation: a modelling study. Lancet Infec Dis 3099: 70121–70124. 10.1016/S1473-3099(12)70121-4 22738893

[20] Chowell G, Viboud C, Munayco CV, Gomez J, Simonsen L, et al (2011) Spatial and temporal characteristics of the 2009 H1N1 influenza pandemic in Peru. PLoS ONE 6: e21287. 21712984 10.1371/journal.pone.0021287 PMC3119673

[21] Azzoni C, Menezes-Filho N, Menezes T, Silveira-Neto R (2000) Geography and Income Convergence Among Brazilian States. IDB Working Paper 122 Available at SSRN. http://dx.doi.org/10.2139/ssrn.1814668.

[22] Brazilian Ministry of Health (2010) Estratégia nacional de vacinação contra o vírus influenza pandêmico (H1N1) 2009. 8 de março a 21 de maio de 2009. Informe Técnico Operacional. Secretaria de Vigilância em Saúde. Departamento de Vigilância Epidemiologica, Brazil.

[23] Shaman J, Goldstein E, Lipsitch M Absolute humidity and pandemic versus epidemic influenza. Am J Epidemiol 173: 127–135. 21081646 10.1093/aje/kwq347 PMC3011950

[24] Alonso WJ, Viboud C, Simonsen L, Hirano EW, Daufenbach LZ, et al (2007) Seasonality of influenza in Brazil: a traveling wave from the Amazon to the subtropics. Am J Epidemiol 165: 1434–1442. 17369609 10.1093/aje/kwm012

[25] Moura FE, Perdigao AC, Siqueira MM (2009) Seasonality of influenza in the tropics: a distinct pattern in northeastern Brazil. Am J Trop Med Hyg 81: 180–183. 19556586

[26] Wu JT, Cowling BJ, Lau EHY, Ho L–M K.M. D, et al (2010 ) School closure and mitigation of pandemic (H1N1) 2009, Hong Kong. Emerg Infect Dis 16: 538–541. 20202441 10.3201/eid1603.091216 PMC3206396

[27] Chowell G, Viboud C, Simonsen L, Miller M, Alonso W (2010) The reproduction number of seasonal influenza epidemics in Brazil,1996–2006. Proc Biol Sci 277: 1857–1866. 20150218 10.1098/rspb.2009.1897 PMC2871867

[28] Steel J, Palese P, Lowen A (2011 ) Transmission of a 2009 pandemic influenza virus shows a sensitivity to temperature and humidity similar to that of an H3N2 seasonal strain. J Virol 85: 1400–1402. 21084485 10.1128/JVI.02186-10 PMC3020521

[29] Nzussouo NT, Michalove J, Diop OM, Njouom R, Monteiro MdL, et al (2012) Delayed 2009 Pandemic Influenza A(H1N1) circulation in West Africa, May 2009– April 2010. J Infect Dis, in press. 10.1093/infdis/jis572 23169954

[30] Chowell G, Bertozzi SM, Colchero MA, Lopez-Gatell H, Alpuche-Aranda C, et al (2009) Severe respiratory disease concurrent with the circulation of H1N1 influenza. N Engl J Med. NEJMoa0904023. 10.1056/NEJMoa0904023 19564633

[31] Charu V, Chowell G, Palacio Mejia L, Echevarría-Zuno S, Borja-Aburto V, et al (2011) Mortality burden of the A/H1N1 pandemic in Mexico: a comparison of deaths and years of life lost to seasonal influenza. Clin Infect Dis 53: 985–993. 21976464 10.1093/cid/cir644 PMC3202315

[32] Shrestha S, Swerdlow D, Borse R, Prabhu V, Finelli L, et al (2011) Estimating the burden of 2009 pandemic influenza A (H1N1) in the United States (April 2009–-April, 2010). Clin Infect Dis 52: S75–82. 21342903 10.1093/cid/ciq012

[33] Blyth C, Kelso A, McPhie K Ratnamohan V, Catton M, et al (2010 ) The impact of the pandemic influenza A(H1N1) 2009 virus on seasonal influenza A viruses in the southern hemisphere. Euro Surveill 15: 19631. 20738990

[34] Wijngaard C, Asten L, Koopmans M, van Pelt W, Nagelkerke N, et al (2012) Comparing pandemic to seasonal influenza mortality: moderate impact overall but high mortality in young children. PLoS ONE 7: e31197. 22319616 10.1371/journal.pone.0031197 PMC3272034

