# Supplementary material for: Were Equatorial Regions Less Affected by the 2009 Influenza Pandemic? The Brazilian Experience
Source: PLoS One. 2012 Aug 1;7(8):e41918. doi: 10.1371/journal.pone.0041918 (PMC3411570; doi:10.1371/journal.pone.0041918)
Supplement: Box S1 — Analysis of latitudinal gradient in pandemic-associated mortality in Brazil in 2009. (DOC) [file pone.0041918.s001.doc]

Schuck-Paim et al. 2012. Were equatorial regions less affected by the 2009 influenza pandemic? The Brazilian experience.

Box S1. There was a significant negative association between latitude and both laboratory-confirmed (*F_1,26_*=20.7, *P*=0.001, *R*^2^_adj_= 0.43) and excess P&I mortality rates (*F_1,26_*=29.9, *P*<0.001, *R*^2^_adj_= 0.53) limited to the period from June to December 2009. Similarly to the analysis for the entire pandemic period (Jun 2009 to May 2010), here too visual inspection of the data suggested that the relationship between laboratory-confirmed mortality and latitude was clearer in the southern regions. Indeed, the effect of the interaction of latitude with a binary indicator of location (coded 0 above latitude 15^o^S and 1 below it) improved the model’s predictive power as measured by a change in *R*^2^_adj_, from *R*^2^_adj_= 0.43 to *R*^2^_adj_= 0.51.
