# Supplementary material for: Were Equatorial Regions Less Affected by the 2009 Influenza Pandemic? The Brazilian Experience
Source: PLoS One. 2012 Aug 1;7(8):e41918. doi: 10.1371/journal.pone.0041918 (PMC3411570; doi:10.1371/journal.pone.0041918)
Supplement: Box S2 — Analysis of the effect socio-demographic indicators on pandemic-associated mortality in Brazil. (DOC) [file pone.0041918.s002.doc]

Schuck-Paim et al. 2012. Were equatorial regions less affected by the 2009 influenza pandemic? The Brazilian experience.

Box S2. Latitude is a proxy variable for several social and demographic factors that may also affect the circulation and transmission dynamics of influenza. As shown in Table S1, population size, demographic density, the proportion of the population living in urban areas, the age structure of the population (as measured the ratio of young adults - 15-55 years of age - to seniors) and the distance from where the first pandemic death was confirmed (Sao Paulo) were all significantly associated to latitude. We explored the effect of these indicators on mortality, and their role on the observed latitudinal gradients on pandemic severity. To this end, we conducted an exploratory analysis using general linear models to determine if the latitudinal gradient on mortality remained significant after accounting for each factor. Given the observed correlation between many of the independent variables and latitude (Table S1), we used the sequential sum of squares to prevent issues of multicollinearity from arising, including the demographic factor tested as the first term in the model and latitude as a second term. This enabled examining the main effects of each demographic variable on mortality, as well as determining the predictive power of latitude over the pandemic mortality burden once the effect of each demographic variable had been accounted for. We next inverted the order of the first and second terms to examine if the demographic indicator had an effect on mortality after controlling for latitude (p-value^2^ on Table S2).

The latitudinal gradient in mortality remained significant even when the effect of each of the socio-demographic indicators was controlled (Table S2, p-value^1^). Although there was a significant association between distance from Sao Paulo and mortality rates, the lack of significance when the effect of latitude is controlled, and the observation that lab-confirmed death rates remained significant when controlling for distance, suggests that this association was likely a by-product of the correlation between distance from Sao Paulo and latitude. The results also show an association between demographic density and mortality (lab-confirmed death rates) when the effect of latitude was controlled. Similarly, the age structure of the population also explained a small proportion of the variance in lab-confirmed death rates when the effects of latitude were controlled. We therefore tested a general linear model which included all socio-demographic factors for which a significant association with mortality was detected (as indicated by p-value^2^ in Table S2). The results indicate that over 70% (*R*^2^_adj_= 0.73) of the variance in laboratory-confirmed death rates was explained by a model including latitude, demographic density, age structure and the binary location indicator. Latitude was the factor most strongly associated to excess mortality in this multivariate model (Table S3). It is worth noticing that age structure and demographic density were found to be associated to mortality only after correcting for latitude: those densely populated areas (within a same latitude) experienced lower rates of mortality. It is possible that the likely better access to nursing care in the denser urban settings contributed to this pattern.
