# Supplementary material for: Were Equatorial Regions Less Affected by the 2009 Influenza Pandemic? The Brazilian Experience
Source: PLoS One. 2012 Aug 1;7(8):e41918. doi: 10.1371/journal.pone.0041918 (PMC3411570; doi:10.1371/journal.pone.0041918)
Supplement: Table S1 — Bivariate correlation matrix showing the direction and magnitude of the association between socioeconomic and demographic indicators and latitude in Brazil. To standardize the distribution of residuals population size, demographic density and age structure were log-transformed. The proportion of the population living in urban areas and the proportion of children in the population were square-root arcsine transformed. (DOC) [file pone.0041918.s003.doc]

Schuck-Paim et al. 2012. Were equatorial regions less affected by the 2009 influenza pandemic? The Brazilian experience.

Table S1.

|  | Population  Size  (log) | Demographic Density  (log) | Distance from  São Paulo | Proportion  Urban Areas (Asin) | Age  Structure (log) | Proportion of Children  (<15 years) |
| --- | --- | --- | --- | --- | --- | --- |
| Latitude | **-.540**** | **-.480*** | **.891**** | **-.585**** | **.518**** | **.834**** |
| Population Size (log) |  | .561** | -.607** | .288 | -.726** | -.684** |
| Demographic Density (log) |  |  | -.560** | .449* | -.629** | -.699** |
| Distance from São Paulo |  |  |  | -.690** | .507** | .836** |
| Proportion Urban areas (Asin) |  |  |  |  | -.064 | -.551** |
| Age Structure (log) |  |  |  |  |  | .740** |
