# Supplementary material for: Were Equatorial Regions Less Affected by the 2009 Influenza Pandemic? The Brazilian Experience
Source: PLoS One. 2012 Aug 1;7(8):e41918. doi: 10.1371/journal.pone.0041918 (PMC3411570; doi:10.1371/journal.pone.0041918)
Supplement: Table S2 — General linear models testing the effect of socio-demographic factors on pandemic-associated mortality in Brazil. The sequential sum of squares is used in all models. In the first column (p-value1) the demographic indicator is the first term in the model, latitude is the second, the binary location factor is the third and the interaction between latitude and the binary indicator is the fourth term. In the second column (p-value2) the order of the first and second term is inverted. Significant p-values1 and p-values2 indicate, respectively, a significant effect of the factor on mortality before and adjusting for latitude. (DOC) [file pone.0041918.s004.doc]

Schuck-Paim et al. 2012. Were equatorial regions less affected by the 2009 influenza pandemic? The Brazilian experience.

Table S2.

|  |  | P&I Excess  Deaths/100,000 | | | Lab-Confirmed  Deaths/100,000 | | |
| --- | --- | --- | --- | --- | --- | --- | --- |
| Factor | Terms | *R^2^* | p-value^1^ | p-value^2^ | *R^2^* | p-value^1^ | p-value^2^ |
| Age | Age Struct. | 0.401 | 0.158 | 0.360 | 0.663 | 0.891 | **0.002** |
| Structure | Latitude |  | **0.000** | **0.000** |  | **0.000** | **0.000** |
|  | Location (Bin) |  | 0.480 |  |  | 0.056 |  |
|  | Location* Latitude |  | 0.341 |  |  | 0.011 |  |
| Population | Population | 0.378 | 0.082 | 0.566 | 0.604 | 0.121 | 0.183 |
|  | Latitude |  | **0.001** | **0.000** |  | **0.000** | **0.000** |
|  | Location (Bin) |  | 0.747 |  |  | 0.008 |  |
|  | Location* Latitude |  | 0.331 |  |  | 0.015 |  |
| Distance | Distance | 0.430 | **0.000** | 0.350 | 0.623 | **0.000** | 0.286 |
|  | Latitude |  | 0.151 | **0.000** |  | **0.003** | **0.000** |
|  | Location (Bin) |  | 0.457 |  |  | 0.001 |  |
|  | Location*Latitude |  | 0.164 |  |  | 0.073 |  |
| Density | Density | 0.395 | 0.283 | 0.274 | 0.727 | 0.387 | **0.000** |
|  | Latitude |  | **0.000** | **0.000** |  | **0.000** | **0.000** |
|  | Location |  | 0.658 |  |  | 0.006 |  |
|  | Location* Latitude |  | 0.428 |  |  | 0.018 |  |
| Urbanization | Urbaniz. | 0.374 | **0.013** | 0.790 | 0.572 | **0.000** | 0.057 |
|  | Latitude |  | **0.003** | **0.000** |  | **0.010** | **0.000** |
|  | Location |  | 0.590 |  |  | 0.049 |  |
|  | Location* Latitude |  | 0.355 |  |  | 0.023 |  |
