# Supplementary material for: Were Equatorial Regions Less Affected by the 2009 Influenza Pandemic? The Brazilian Experience
Source: PLoS One. 2012 Aug 1;7(8):e41918. doi: 10.1371/journal.pone.0041918 (PMC3411570; doi:10.1371/journal.pone.0041918)
Supplement: Table S3 — Multivariate model testing the effect of latitude, age structure and demographic density on pandemic death rates that were laboratory-confirmed ( R 2adj = 0.73) in Brazil. (DOC) [file pone.0041918.s005.doc]

Schuck-Paim et al. 2012. Were equatorial regions less affected by the 2009 influenza pandemic? The Brazilian experience.

Table S3.

| Model Term | Type I Sum of Squares | D.F. | F-ratio | P-value |
| --- | --- | --- | --- | --- |
| Latitude | 2.671 | 1 | 38.641 | 0.000 |
| Age Structure | 1.074 | 1 | 15.537 | 0.001 |
| Demographic Density | 0.542 | 1 | 7.835 | 0.011 |
| Location (Binary) | 0.431 | 1 | 6.238 | 0.021 |
| Location (Binary) * Latitude | 0.490 | 1 | 7.082 | 0.015 |
| Error | 1.452 | 21 |  |  |
